# Supplementary material for: Image quality of abdominal ultrasonography after esophagogastroduodenoscopy is preserved by using carbon dioxide insufflation: A non-inferiority test in the same subject
Source: PLoS One. 2022 Sep 29;17(9):e0275257. doi: 10.1371/journal.pone.0275257 (PMC9521841; doi:10.1371/journal.pone.0275257)
Supplement: Attachment — Submitted filename: PLOS.docx [file pone.0275257.s002.docx]

Reviewer comments on the manuscript titled**,” Image quality of abdominal ultrasonography after esophagogastroduodenoscopy is preserved by using carbon dioxide insufflation: A non-inferiority test in the same subject**”.

It is always considered better if a patient can undergo USG abdomen (AUSG) and Esophagogastroduodenoscopy (EGD) on the same day one after another. This would reduce the problems associated with the delay in the allocation of dates for the two procedures on the same subject. However due to the air inside the stomach and the duodenum, following EGD, the USG abdomen cannot be done on account of poor visibility. It is hypothesized that this can be overcome if CO2 is used as the insufflating agent in place of air. In order to prove this hypothesis, the authors have pursued the study. The strengths of the study are 1) the adequate sample size of the study subjects and 2) the blinding of the interpreters of the USG images done by three experienced USG operators. I would however like to obtain a few responses from the authors regarding the following issues:

1. Figure 1 does not have any title, legend or label. The X-axis and the Y-axis should be labelled.
2. The authors have mentioned the study type as a prospective one. However, there hasn’t been any follow-up of the subjects. Therefore it is a cross-sectional study and not a prospective one.
3. USG of an organ is an operator-dependent procedure. That some of the images following EGD appeared better to the interpreters as compared to the images done before EGD emphasizes this fact only. I would request the authors to comment on this fact with reference to the results of the study.
4. P values with confidence intervals were not demonstrated while comparing the USG image quality scores of the different organs before and after EGD. I would request the authors to provide that.
5. There are a few typographical and grammatical errors which the authors need to correct. These are as mentioned below.

Line 89: Insert “and” between AUS and EGD.

Line 97: “sonologists was” should be” sonologists were”.

Line 120: “was” should be “were”

Line 121: “as to” should be removed.

Line 153: “mean” should be “the mean”

Line 166: “considered to be” should be” is considered to be”.

Line 214: “require” should be” requires.”

Line 247: for “a” statistical study.

Line 248: samples “was” should be “were”

Line 270: is same should be is “the” same.

Line 287 image quality should be “the” image quality.
