# Supplementary material for: Image quality of abdominal ultrasonography after esophagogastroduodenoscopy is preserved by using carbon dioxide insufflation: A non-inferiority test in the same subject
Source: PLoS One. 2022 Sep 29;17(9):e0275257. doi: 10.1371/journal.pone.0275257 (PMC9521841; doi:10.1371/journal.pone.0275257)
Supplement: Attachment — Submitted filename: Response to Reviewers 2nd. revise.docx [file pone.0275257.s004.docx]

**Point-by-point responses to the Editor and Reviewers**

**Reviewer #1**

Comment: The authors have responded to my questions regarding the manuscript titled, “Image quality of abdominal ultrasonography after esophagogastroduodenoscopy is preserved by using carbon dioxide insufflation: A non-inferiority test in the same subject” satisfactorily.
There was however one comment to which I felt the authors did not explain clearly. This was the response to comment no 3. The authors said they did not conclude that the USG images improved after EGD using CO_2_ insufflation. Indeed, they did not conclude in that manner but this is also true that they wrote about their observation that the image quality improved in some patients after EGD using CO_2_ insufflation (page 14, lines 287,288) which I feel has no scientific explanation rather than the inherently subjective nature (operator dependence) of the investigation under question. This factor was bound to affect the assessment of the USG images in the study which was assessed based on the four-point Likert scale. However, they have rightly concluded that it is misleading to state that CO_2_ insufflation improves AUS image quality even if this does occur in some patients. I feel that in the absence of a validated pre-existing tool for assessment of the differences in the quality of the two USG images done on the same subject in a short time gap, this paper has merit to be published, provided the statistical part (assessed by reviewer 2) is sound enough.

Response: We believe that the sentence “In the present study, image quality also improved in some patients post-EGD AUS (Fig 4), but for other patients the quality worsened, so it is misleading to state that CO_2_ insufflation improves AUS image quality even if this does occur in some patients” is necessary to disprove the claims of previous papers (reference 4) and to negate the hypothesis that CO_2_ insufflation could improve image quality, which we had expected at the time of planning our study and is of interest to the readers. Thank you for your valuable comments.

**Reviewer #2**

Comment: In my previous review, I raised 4 major issues and 3 specific points. All specific points have been addressed and the authors have provided reasonable answers to major issues 2-4 (thanks). However, I can't accept their answer to major issue 1.

From the answer to specific point no 2, I understand the depiction rate was not computed but it was summarized by the levels of a Linkert scale. Under this setting, two important points raised in my previous review are still unsolved, namely
1) mean differences between Linkert-based scores are not mathematically sound (ordinal variables are better analyzed by quantiles) and, as a result, they are difficult to interpret
2) Linkert-based scores are obviously not normally distributed and, as a result, conventional confidence intervals are inappropriate.

The authors agree with me on these two points, but they claim that their methods are robust with respect to the violations of these assumptions, citing reference 10. It is possible that some methods of the paper are robust, but robustness must be quantified. Unfortunately, I don’t see a simple way to justify robustness under point 1 above, because, as the authors say, we don’t have the continuous value of the depiction rate. Point 2 would instead be addressed by an appropriate simulation study, which is the standard approach to evaluate robustness; citing reference 10 is not enough because the degree of robustness varies from one study to another.

I still believe that trying to prove robustness is not the easiest approach in this study. Acknowledging the qualitative nature of the Linkert scale and using multinomial-based methods (as I suggested in my previous review) is much simpler. Furthermore, robustness is usually invoked when there are not standard methods to avoid assumptions violation. However, multinomial methods are standard and available in many statistical packages.

Response: Our present study was designed and conducted based on statistical methods described in “Reviews and Commentary” of Radiology (reference 5), the most authoritative journal in diagnostic imaging. In clinical research, some flexibility is required to find a compromise between limitations in sample collection (in the evaluation of image quality, the limitation is that there is no method other than the Likert scale) and statistical correctness. In this review, the multinomial regression method that you are recommending is not mentioned, whereas a non-inferiority test using the mean difference is recommended to demonstrate non-inferiority of the outcome of one group to the outcome of another group, because non-inferiority cannot be proven correctly by multinomial regression methods. Furthermore, use of the mean difference between ordinal variables, such as Likert-based scores or grading scores is accepted. In fact, a study that is mentioned in this review used a non-inferiority test using the mean difference between grading scores. Please see the contents in the Abstract of that study below.

Hausleiter J, Martinoff S, Hadamitzky M, Martuscelli E, Pschierer I, Feuchtner GM, et al. Image quality and radiation exposure with a low tube voltage protocol for coronary CT angiography results of the PROTECTION II Trial. JACC Cardiovasc Imaging. 2010;3(11):1113-23.

Abstract

Objectives: The purpose of this study was to evaluate image quality and radiation dose using a 100 kVp tube voltage scan protocol compared with standard 120 kVp for coronary computed tomography angiography (CTA).

Background: Concerns have been raised about radiation exposure during coronary CTA. The use of a 100 kVp tube voltage scan protocol effectively lowers coronary CTA radiation dose compared with standard 120 kVp, but it is unknown whether image quality is maintained.

Methods: We enrolled 400 nonobese patients who underwent coronary CTA: 202 patients were randomly assigned to a 100 kVp protocol and 198 patients to a 120 kVp protocol. The primary end point was to demonstrate noninferiority in image quality with the 100 kVp protocol, which was assessed by a 4-point grading score (1 = nondiagnostic, 4 = excellent image quality). For the noninferiority analysis, a margin of -0.2 image quality score points for the difference between both scan protocols was pre-defined. Secondary end points included radiation dose and need for additional diagnostic tests during follow-up.

Results: The mean image quality scores in patients scanned with 100 kVp and 120 kVp were 3.30 ± 0.67 and 3.28 ± 0.68, respectively (p = 0.742); image quality of the 100 kVp protocol was not inferior, as demonstrated by the 97.5% confidence interval of the difference, which did not cross the pre-defined noninferiority margin of -0.2. The 100 kVp protocol was associated with a 31% relative reduction in radiation exposure (dose-length product: 868 ± 317 mGy × cm with 120 kVp vs. 599 ± 255 mGy × cm with 100 kVp; p < 0.0001). At 30-day follow-up, the need for additional diagnostic studies did not differ (13.4% vs. 19.2% for 100 kVp vs. 120 kVp, respectively; p = 0.114).

Conclusions: A coronary CTA protocol using 100 kVp tube voltage maintained image quality, but reduced radiation exposure by 31% as compared with the standard 120 kVp protocol. Thus, 100 kVp scan protocols should be considered for nonobese patients to keep radiation exposure as low as reasonably achievable. (Prospective Randomized Trial on Radiation Dose Estimates of Cardiac CT Angiography in Patients Scanned With a 100 kVp Protocol [PROTECTION II]; NCT00611780).

The sentence in your comment “as the authors say, we don’t have the continuous value of the depiction rate” appears to potentially be the result of a misunderstanding. We stated that “A method for evaluating the depiction rate of organs in AUS as continuous variables has not been established, and previous studies have used a Likert scale. To resolve this problem, we developed a more objective scale by replacing the abstract evaluation with the depiction rate as an interval scale more suitable for statistical analysis”. Again, the Likert scale we used is not an ordinal scale like the conventional Likert scale. It is an interval scale, even if it cannot be measured finely. Variables measured by the interval scale are continuous variables. Because it seems that the use of the word “Likert scale” is misleading, we have changed it to “Likert-like scale” in the revised manuscript.

There also appears to be a potential misunderstanding regarding reference 10. The paper (reference 10) is not just a single study. This paper shows that many studies, dating back to the 1930s, consistently show that parametric statistics are robust with respect to violations of many assumptions. Please see the contents in the Abstract below.

Reference 10. Norman G. Likert scales, levels of measurement and the "laws" of statistics. Adv Health Sci Educ Theory Pract. 2010;15(5):625-32. doi: 10.1007/s10459-010-9222-y.

Abstract

Reviewers of research reports frequently criticize the choice of statistical methods. While some of these criticisms are well-founded, frequently the use of various parametric methods such as analysis of variance, regression, correlation are faulted because: (a) the sample size is too small, (b) the data may not be normally distributed, or (c) The data are from Likert scales, which are ordinal, so parametric statistics cannot be used. In this paper, I dissect these arguments, and show that many studies, dating back to the 1930s consistently show that parametric statistics are robust with respect to violations of these assumptions. Hence, challenges like those above are unfounded, and parametric methods can be utilized without concern for ‘‘getting the wrong answer’’.

Although our present study is a clinical one with limited collection of samples with fine measurements, we believe that our analysis is based on recommended statistical methods by the authoritative journal, and that we have made every effort to be statistically accurate.

Changes 1: The following has been added to the revised manuscript on lines 113-119, and the revisions are shown in red.

In this study, we developed a more objective scale by replacing the abstract evaluation with the depiction rate as an interval scale suitable for statistical analysis [10]. All saved AUS moving images, which were marked only with a study code, were randomized and then evaluated. To reduce systematic error, four expert reviewers board-certified by the Japanese Society of Gastroenterology rated image quality in all moving images using a four-point Likert scale: 1 - depiction rate of 0%–24%, 2 - 25%–49%, 3 - 50%–74%, 4 - 75%–100%.

→

In this study, we developed a more objective scale by replacing the abstract evaluation with the depiction rate as an interval scale suitable for statistical analysis [10]. All saved AUS moving images, which were marked only with a study code, were randomized and then evaluated. To reduce systematic error, four expert reviewers board-certified by the Japanese Society of Gastroenterology rated image quality in all moving images using a four-point Likert-like scale as an interval scale: 1 - depiction rate of 0%–24%, 2 - 25%–49%, 3 - 50%–74%, 4 - 75%–100%.

Changes 2: The following has been added to the revised manuscript on lines 28-30, and the revisions are shown in red.

All saved AUS moving images were randomized and imaging quality was evaluated at 16 organs using a four-point Likert scale that divides the depiction rate by 25%.

→

All saved AUS moving images were randomized and imaging quality was evaluated at 16 organs using a four-point Likert-like scale that divides the depiction rate by 25%.

Changes 3: The following has been added to the revised manuscript on lines 252-254, and the revisions are shown in red.

Because the present –0.40-point margin of a four-point Likert scale is quite small compared with these reports, it is hard to say that our margin is too generous.

→

Because the present –0.40-point margin of a four-point Likert-like scale is quite small compared with these reports, it is hard to say that our margin is too generous.

Changes 4: The following has been added to the revised manuscript on lines 393-394, and the revisions are shown in red.

S1 Table. Dataset of examinees' characteristics and Likert scales evaluated by the four reviewers for 16 organs in thirty subjects.

→

S1 Table. Dataset of examinees' characteristics and Likert-like scales evaluated by the four reviewers for 16 organs in thirty subjects.
